# Dissolution or Growth of a Liquid Drop via Phase-Field Ternary Mixture Model Based on the Non-Random, Two-Liquid Equation

**DOI:** 10.3390/e20020125

**Published:** 2018-02-14

**Authors:** Andrea Lamorgese, Roberto Mauri

**Affiliations:** DICI/University of Pisa, Largo Lazzarino 1, 56122 Pisa, Italy

**Keywords:** emulsions, drops, liquid–liquid phase transitions, diffusion, nonequilibrium thermodynamics

## Abstract

We simulate the diffusion-driven dissolution or growth of a single-component liquid drop embedded in a continuous phase of a binary liquid. Our theoretical approach follows a diffuse-interface model of partially miscible ternary liquid mixtures that incorporates the non-random, two-liquid (NRTL) equation as a submodel for the enthalpic (so-called excess) component of the Gibbs energy of mixing, while its nonlocal part is represented based on a square-gradient (Cahn-Hilliard-type modeling) assumption. The governing equations for this phase-field ternary mixture model are simulated in 2D, showing that, for a single-component drop embedded in a continuous phase of a binary liquid (which is highly miscible with either one component of the continuous phase but is essentially immiscible with the other), the size of the drop can either shrink to zero or reach a stationary value, depending on whether the global composition of the mixture is within the one-phase region or the unstable range of the phase diagram.

## 1. Introduction

We present results of phase-field simulations of isothermal dissolution or growth of a single-component liquid drop embedded in the continuous phase of a binary host liquid, as a function of system location in the phase diagram for the ternary mixture. Our attention in this work will be restricted to liquid-liquid systems, although it should be acknowledged that the dissolution or growth of gas bubbles in a gas-liquid system has been addressed in the literature many times, since the pioneering work by Epstein and Plesset [1], who predicted a $t^{1/2}$ shrinkage dynamics for a gas bubble in an undersaturated liquid-gas mixture, solely based on an ideal mixture assumption together with Fick’s law invoked to describe the species mass flux in terms of local concentration gradients. In fact, over the past few decades, much less attention has been directed to liquid-liquid systems, and only recently an extension of the Epstein-Plesset theory has been proposed [2,3] that addresses multicomponent liquid drop dissolution into a second immiscible (host) liquid. Subsequently, the approach advocated by Su and Needham [3] was questioned by Chu and Prosperetti [4], who reported the first physically sound model of multicomponent liquid drop dissolution into a host (immiscible) liquid, showing that the dissolution rate follows from the chemical potential of each component at the drop interface, assuming that the interface is in a state of local thermodynamic equilibrium (UNIQUAC-based activity coefficients were also employed in their model to account for the interaction between each of the constituent species of a binary drop and the bulk liquid).

Although a number of theoretical, numerical, and experimental investigations of multicomponent liquid drop dissolution have now been made, none of those previous studies, however, have addressed the dependence of the transient drop dynamics on the global mixture composition in the ternary phase diagram. Indeed, since inherent in all of the aforementioned works is an assumption of infinite expanse for the host liquid (in addition to a sharp-interface assumption for all two-phase interfaces), the global composition of the bulk liquid mixture is essentially fixed and cannot be accounted for (as a parameter). In fact, this dependence has been addressed very recently by Lamorgese and Mauri [5], who showed an isothermal phase-field ternary mixture model in application to an isolated, single-component (resp. two-component) drop embedded in a continuous phase of a binary (resp. single-component) liquid as it grows or dissolves depending on the global state of the system in the ternary phase diagram. They reported both reabsorbing and unstable drop cases, most of which could be characterized as trivial from a dynamical point of view, i.e., in those cases, the radius of the drop simply increases or decreases to a stationary value with no other interesting phenomena taking place; however, they also found an unstable case showing a drop whose transient growth is accompanied by a spinodal wave (i.e., a compositional disturbance propagating outwards from the center of the drop, converting an otherwise uniform composition into a collection of small drops). Now, although the appearance of an array of droplets behind the front of a spinodal wave represents a novel finding from that work, it should be acknowledged that the results of that work were based on the premise that the ternary liquid mixtures of interest could be described via the one-parameter Margules correlation (i.e., Porter’s equation [6]), which is the simplest excess free energy model one can use for a nonideal liquid mixture. However, for strongly nonideal mixtures (particularly for partially immiscible systems), the two-parameter Margules correlation may simply be unable to provide an accurate representation of experimental activity coefficient data, even when great care is exercised in the data reduction needed to obtain the adjustable parameters. Therefore, in this work, we intend to further address an isolated single-component liquid drop embedded in a continuous phase of a binary liquid by means of a diffuse-interface model of phase transition in ternary liquid mixtures that relies on a more accurate representation of the excess part of the Gibbs energy of mixing, so that the resulting model should be better suited to correlate ternary liquid-liquid equilibrium data for a homogeneous mixture and simultaneously predict (with improved accuracy as compared to the two-parameter Margules correlation) ternary phase diagrams from binary-type information. In this work, we restrict our attention to the non-random, two-liquid (NRTL) model [7], which represents the simplest improvement over the two-parameter Margules correlation based on the local composition concept [7,8,9].

It is worth reiterating, regarding our choice of modeling ternary liquid mixtures within the frame of a diffuse-interface description, that in addition to its ability to account for nonideal mixture thermodynamics, the diffuse-interface model offers an alternative approach to the classical sharp-interface description of multiphase systems, avoiding physical property and dynamic variable discontinuities at geometrically complex interfaces. Instead, it treats the interfaces as having nonzero thickness, and, as a result, all physical quantities vary continuously across the interfacial volume, a layer of finite (sub-micron-scale) thickness. Another advantage of the diffuse-interface model is that its governing equations are valid for equilibrium and general nonequilibrium conditions alike, while inherent in its formulation is a (nonequilibrium) surface tension that arises as a result of weakly nonlocal effects in the thermodynamic potentials [10,11] (i.e., such potentials also depend on the spatial gradients of the order parameter(s)). This is in contrast to a sharp-interface description, wherein both assumptions of a local thermodynamic equilibrium and constant surface tension (which are normally incorporated into sharp-interface-based numerical models) are expected to fail in far-from-equilibrium situations. (This point is elaborated on further in Reference [11], where a comparative analysis of phase-field and Gibbs dividing surface models is presented).

The remainder of this paper is laid out as follows. In the following, we present a quick summary of numerical methods after a brief review of the governing equations for our phase-field formulation. Then, we show the results of 2D phase-field simulations of diffusion-driven dissolution or growth for an isolated liquid drop embedded in a continuous phase of another liquid as a function of system location in the ternary phase diagram. Finally, at the end, a few conclusions are drawn.

## 2. Model Description

The derivation of the governing equations for regular ternary liquid mixtures with partially miscible components within a diffuse-interface description has been presented elsewhere [5,12]. Herein, to emphasize that inherent in the equations shown previously is a nonequilibrium thermodynamics modeling assumption for the species diffusive fluxes, we show again below the (nonreactive) species conservation equations in the absence of convection:

(1) $$\begin{array}{cc} \frac{\partial x_{1}}{\partial t} & =-\nabla \cdot \frac{J_{1}}{c} \end{array}$$

(2) $$\begin{array}{cc} \frac{\partial x_{2}}{\partial t} & =-\nabla \cdot \frac{J_{2}}{c} \end{array}$$

where $x_{i}=c_{i}/c$ is the mole fraction for the *i*th component ($c_{i}$ being its mole density) in a regular ternary mixture, while $J_{i}/c$ denotes a diffusive volumetric flux ($c=\sum_{k}c_{k}$ being the total mole density, assumed to be constant herein). Note that, in general, the diffusive volumetric fluxes for a ternary system can be represented in the form:

(3) $$\begin{array}{cc} \frac{J_{1}}{c} & =x_{1}x_{2}\left(v_{1}-v_{2}\right)+x_{1}x_{3}\left(v_{1}-v_{3}\right) \end{array}$$

(4) $$\begin{array}{cc} \frac{J_{2}}{c} & =x_{2}x_{1}\left(v_{2}-v_{1}\right)+x_{2}x_{3}\left(v_{2}-v_{3}\right) \end{array}$$

with $v_{i}$ denoting the *i*th species velocity. We now introduce the assumption (that is normally adopted in nonequilibrium thermodynamics modeling [13,14]) that the diffusive components of the species molar fluxes can be represented as the negative gradient of a generalized chemical potential, i.e., $v_{i}-\;\;\;v^{*}=-D\nabla {\tilde{\mu }}_{i}$, with *D* denoting an as yet unspecified diffusion coefficient ($v^{*}$ being the mole-averaged velocity); this is then equivalent to assuming that

(5) $$v_{i}-v_{j}=-D_{ij}\nabla {\tilde{\mu }}_{ij}$$

where $D_{ij}=D_{ji}$ denotes a binary diffusivity, while ${\tilde{\mu }}_{ij}\equiv {\tilde{\mu }}_{i}-{\tilde{\mu }}_{j}$ denote generalized chemical potential differences (further addressed below). In fact, when each velocity difference in Equations (3) and (4) is substituted for from Equation (5), we see that the resulting constitutive equations for the diffusive volumetric fluxes (i) satisfy the Onsager reciprocity relation [5], and (ii) are consistent with a nonnegative entropy production rate

(6) $$\frac{\sigma_{s}}{R}=-\frac{J_{1}}{c}\cdot \nabla {\tilde{\mu }}_{13}-\frac{J_{2}}{c}\cdot \nabla {\tilde{\mu }}_{23}=\sum_{i}\sum_{j>i}D_{ij}x_{i}x_{j}{\left|\nabla {\tilde{\mu }}_{ij}\right|}^{2}$$

in the entropy equation for a purely diffusive ternary system (i.e., in the absence of convection). In what follows, we further assume equality of all binary diffusivities; as a result, the species conservation equations (Equations (1) and (2)) can be rearranged to the form [5]:

(7) $$\begin{array}{cc} \frac{\partial x_{1}}{\partial t} & =\nabla \cdot \left\{-x_{1}x_{2}\nabla {\tilde{\mu }}_{23}+x_{1}\left(1-x_{1}\right)\nabla {\tilde{\mu }}_{13}\right\} \end{array}$$

(8) $$\begin{array}{cc} \frac{\partial x_{2}}{\partial t} & =\nabla \cdot \left\{-x_{1}x_{2}\nabla {\tilde{\mu }}_{13}+x_{2}\left(1-x_{2}\right)\nabla {\tilde{\mu }}_{23}\right\} \end{array}$$

These equations have been scaled based on a characteristic length *a* (which is representative, on the mesoscale, of the typical interface thickness at local equilibrium) and the diffusive time $a^{2}/D$, where *D* denotes the (same) binary diffusivity for all component pairs. The generalized chemical potential for the *i*th component, ${\tilde{\mu }}_{i}=\mu_{i}^{th}+\mu_{i}^{nl}$, is the sum of a thermodynamic part

(9) $$\mu_{i}^{th}=\frac{1}{RT}{\left(\frac{\partial cg^{th}}{\partial c_{i}}\right)}_{T,P,c_{j\neq i}}$$

which is defined as a partial molar quantity [9] when the mixture is homogeneous, and a nonlocal part

(10) $$\mu_{i}^{nl}=-\frac{1}{RT}\nabla \cdot {\left(\frac{\partial cg^{nl}}{\partial \nabla c_{i}}\right)}_{T,P,\nabla c_{j\neq i}}$$

which arises as a result of spatial inhomogeneities in the composition [12]. We note that the nonlocal part of the chemical potential for the *i*th species is the result of a Cahn-Hilliard-type modeling for the nonlocal component of the Gibbs energy, i.e., $\tilde{g}=g^{th}+g^{nl}$, where [12]

(11) $$g^{nl}=-RT\frac{a^{2}}{2}\left(\nabla x_{1}\cdot \nabla x_{2}+\nabla x_{1}\cdot \nabla x_{3}+\nabla x_{2}\cdot \nabla x_{3}\right)$$

Now, at variance with the derivations in our previous works [5,12], for modeling the enthalpic (so-called excess) part of the thermodynamic (molar) Gibbs energy of mixing

(12) $$g^{th}-\left(g_{1}x_{1}+g_{2}x_{2}+g_{3}x_{3}\right)=\Delta g^{th}=\Delta g^{id}+\Delta g^{ex}$$

we have recourse herein to the NRTL equation [6,7,9], i.e.,

(13) $$\frac{\Delta g^{ex}}{RT}=\sum_{i=1}^{n}x_{i}\frac{L_{i}}{M_{i}}\;(n=3)$$

while, as is well known, the ideal (or entropic) part is given by [15]

(14) $$\Delta g^{id}=RT\left(x_{1}\ln x_{1}+x_{2}\ln x_{2}+x_{3}\ln x_{3}\right)$$

We have employed standard nomenclature [6,7,9] in Equation (13), i.e.,

(15) $$\begin{array}{cccc} L_{i} & =\sum_{k=1}^{n}x_{k}\tau_{ki}G_{ki},\; & G_{ij} & =\exp\left\{-\alpha_{ij}\tau_{ij}\right\} \end{array}$$

(16) $$\begin{array}{cccc} M_{i} & =\sum_{k=1}^{n}x_{k}G_{ki},\; & \tau_{ij} & =\frac{\Delta g_{ij}}{RT}\left(1-\delta_{ij}\right) \end{array}$$

where $\alpha_{ij}=\alpha_{ji}$. These relations then show that Equation (13) for a ternary system contains nine adjustable binary parameters (i.e., three α’s in addition to six (off-diagonal) $\Delta g_{ij}$ terms). Based on Equation (13), we obtain the following activity coefficients [6,7,9]:

(17) $$\ln\gamma_{i}=\frac{L_{i}}{M_{i}}+\sum_{j=1}^{n}\frac{x_{j}G_{ij}}{M_{j}}\left(\tau_{ij}-\frac{L_{j}}{M_{j}}\right)$$

Finally, we show below the generalized chemical potential differences that are needed to evaluate the diffusive volumetric fluxes:

(18) $$\begin{array}{cc} {\tilde{\mu }}_{13} & =\frac{g_{1}-g_{3}}{RT}+\ln\frac{\gamma_{1}x_{1}}{\gamma_{3}x_{3}}-\frac{a^{2}}{2}\nabla^{2}\left(x_{1}-x_{3}\right) \end{array}$$

(19) $$\begin{array}{cc} {\tilde{\mu }}_{23} & =\frac{g_{2}-g_{3}}{RT}+\ln\frac{\gamma_{2}x_{2}}{\gamma_{3}x_{3}}-\frac{a^{2}}{2}\nabla^{2}\left(x_{2}-x_{3}\right) \end{array}$$

where $g_{i}$ denotes the (molar) Gibbs energy of the *i*th pure species. These relations, together with the equalities ${\tilde{\mu }}_{ij}={\tilde{\mu }}_{ik}+{\tilde{\mu }}_{kj}$ and ${\tilde{\mu }}_{ij}=-{\tilde{\mu }}_{ji}$, define all chemical potential differences for a ternary system.

In conclusion, it is worth reiterating that Equations (7) and (8) constitute a system of fourth-order equations, which represents a generalization of the classical Cahn-Hilliard equation to describe phase separation in binary mixtures [11,16,17,18,19,20,21,22,23].

## 3. Numerical Methods

Since we are not interested in the effect of nontrivial boundary conditions (such as, e.g., when a contact line arises at a solid boundary) on the temporal evolution of the concentration fields, herein we will simulate the species balance equations above using periodic boundary conditions. This enables a straightforward pseudospectral spatial discretization of the governing equations. In fact, although we attempted to march forwards in time the governing equation system using the Cash-Karp Runge-Kutta scheme [24,25,26] after a pseudospectral spatial discretization, based on numerical tests we found that our previous temporal integration scheme [26] could not surmount the increase in stiffness incurred when NRTL is employed in place of the one-parameter Margules correlation as an excess-free energy model. With our pseudospectral code, we found that the stiffness of the system could not be surmounted by increasing the order of the temporal integrator (specifically, this observation applies to the case wherein the Cash-Karp Runge-Kutta scheme is replaced with the Runge-Kutta-Verner 7(6) scheme [27]). However, with the NRTL model in place, we also found that assuming an explicit, adaptive temporal scheme, issues of numerical instability and step-size underflow (found with the pseudospectral discretization) disappeared when the spatial discretization was changed from pseudospectral to finite volume. In fact, using a standard finite-volume discretization, we saw that although the Cash-Karp Runge-Kutta scheme failed due to a step-size underflow, with the adaptive Runge-Kutta-Verner 6(5) scheme [27] the time-marching achieved a stable integration. Again, this observation was confirmed in another test case with the adaptive Runge-Kutta-Verner 7(6) scheme. Finally, we chose this last temporal scheme (in conjunction with a finite-volume discretization) in our production runs for calculating the results presented below.

## 4. Results and Discussion

We now describe numerical results from 2D phase-field simulations of dissolution and growth of an isolated drop of pure component 1, embedded in a continuous phase of highly miscible components 2 and 3 (having a uniform initial composition $x_{2}=x_{3}=0.5$). Specifically, we consider a benzene-acetonitrile-water mixture for which NRTL parameters have been listed by Castillo and Grossmann [28], allowing phase-diagram calculations for that mixture at T=300K and T=333K. (We show in Table 1 those parameters for the reader’s convenience).

**Figure 1:**
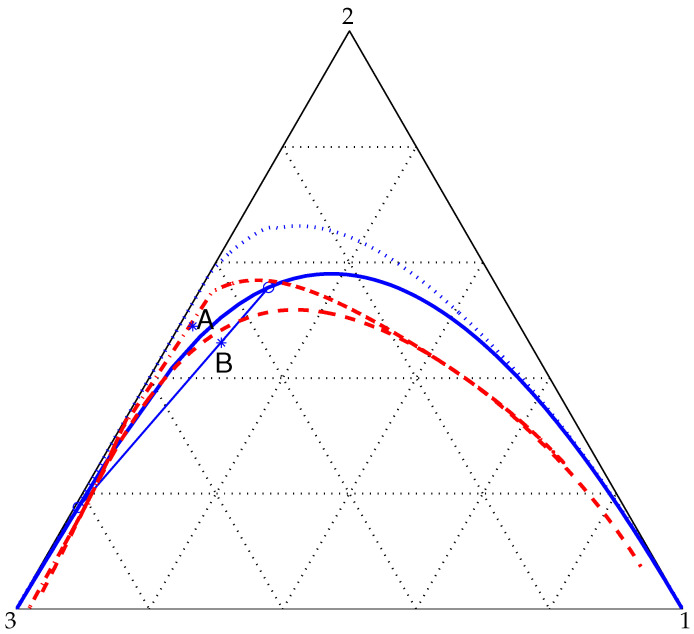
Phase diagram for a (1)benzene-(2)acetonitrile-(3)water mixture at T=333K using NRTL as an excess-free energy model. The coexistence and spinodal curves are shown solid and dashed, respectively. A and B (also referred to as cases A and B in the text) denote equilibrium states, respectively, in the one-phase (stable) and in the two-phase (unstable) regions. The tie-line through point B is also shown. Finally, also included in the diagram are the binodal (dotted) and the spinodal (dot-dashed) curves for the same mixture as above at T=300K.

The liquid mixture of interest herein can also be described as consisting of soluble components 2 (acetonitrile) and 3 (water), also known as the native and primary solvents, after addition of a modifier (benzene) which is insoluble with either the native or the primary solvent. (As far as the binodal and spinodal curves in Figure 1 are concerned, this ternary system is qualitatively similar to the toluene-acetonitrile-water mixture investigated experimentally by Gupta et al. [29] and numerically by Lamorgese and Mauri [5] with the one-parameter Margules correlation).

The phase diagram for this ternary system at T=333K is shown in Figure 1. Note that all ternary liquid-liquid equilibria shown in Figure 1 have been calculated efficiently using a γ-γ method [6] that leverages the Rachford-Rice algorithm [30]. Also, the spinodal curves in Figure 1 have been computed by finding zeros to the Hessian determinant (shown in the Appendix A), which follows from our choice of NRTL as an excess-free energy model. At this point, it is clear that system location in the phase diagram can be adjusted based on the initial radius of the drop [5]. Using a small radius of 20a at the initial time, system location corresponds to point A in Figure 1. (Incidentally, the global composition for a mixture located at A is $x_{A}=\left(0.02,0.489,0.491\right)$).

**Figure 2:**
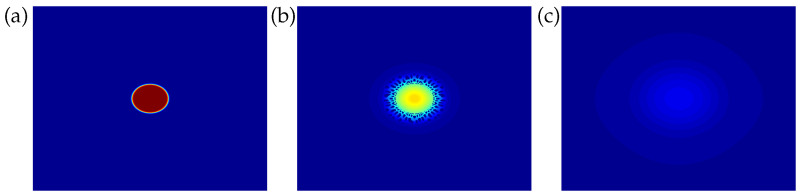
Snapshots of $x_{1}$ mole fraction at different (nondimensional) times (**a**) t=0, (**b**) $t=0.1\;t^{*}$, (**c**) $t=0.99\;t^{*}$ ($t^{*}$ being the instant in time corresponding to a vanishing drop size) from phase-field simulation of a reabsorbing drop (case A in Figure 1) on a $512^{2}$ grid. The initial radius of the drop is 20a.

**Figure 3:**
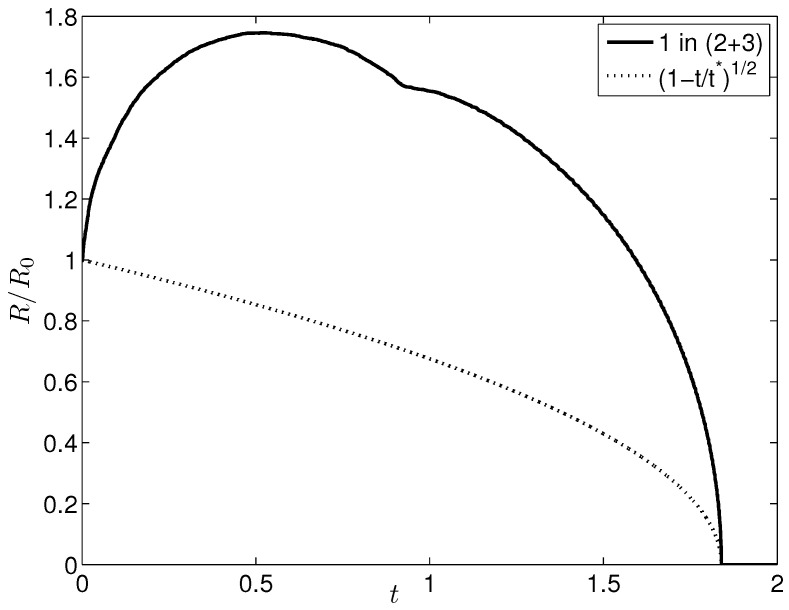
Drop radius (made dimensionless with the initial drop radius) vs. (nondimensional) diffusive time from phase-field simulation of a reabsorbing drop (case A). At time zero, an isolated drop (consisting of pure component 1) is embedded in a two-component (2 + 3) liquid. The mixture is (1)benzene-(2)acetonitrile-(3)water at a uniform temperature T=333K. A $t^{1/2}$ shrinking dynamics (based on the Epstein-Plesset theory [1]) is also shown (dotted).

With this initial condition, we ran a test case (denoted as case A) and checked that, after an initial transient, such a drop eventually dissolves into the continuous phase as the mixture is located at a stable equilibrium state in the one-phase region (representative snapshots from this reabsorbing drop simulation are shown in Figure 2). This case, too, is documented in Figure 3, showing an increase-decrease behavior in the drop radius versus time dependence, which had already been observed in our previous study of diffusion-driven dissolution or growth of a liquid drop [5] based on the one-parameter Margules correlation.

Note that in both simulation cases reported herein all lengths and times were scaled based on $L=\frac{N}{2}a$ (since our computational cell size was chosen equal to a/2), with *N* denoting the number of cells in each coordinate direction, and $L^{2}/D$, respectively, with *L* denoting the periodicity length of the computational domain. Consequently, based on our choice of N=512, we see that $\frac{a^{2}}{D}\approx 1.5\times 10^{-5}$ the unit of time in Figure 3. It should also be acknowledged that, although our simulation results are not affected by the actual values for *a* and *D* (since they drop out of the dimensionless species balance equations), for typical liquid binary mixtures at $20\;{}^{{}^{\circ}}C$ one finds a≈0.1μm and $D\approx 10^{-5}\;{\mathrm{cm}}^{2}/s\;$ [29,31,32,33,34].

Returning to case A above, considering (i) the similarity between the phase diagram in Figure 1 and that for a mixture of toluene-acetonitrile-water (based on the one-parameter Margules correlation and employed for modeling a liquid drop of pure toluene embedded in a continuous phase of acetonitrile+water [5]), and (ii) that the global states of the system (i.e., for case A above and case 2A from our previous study [5]) are similarly located within each (respective) phase diagram, we argue that case A (considered herein), too, can be explained in terms of a fast time scale for component 2 to diffuse into the drop, while component 1 diffuses out of the drop in a time that is approximately twice as long. Also shown in Figure 3 is a $t^{1/2}$ shrinking dynamics (based on the Epstein-Plesset theory [1]), which turns out to be at all times far from the NRTL-based reabsorbing drop case of the present discussion. This is not surprising given that the Epstein-Plesset theory is based on an ideal solution assumption, while, obviously, the NRTL-based modeling presented herein purports to account for (possibly strong) deviations from ideal solution behavior. In particular, at variance with the drop radius versus time dependence based on the one-parameter Margules correlation [5], a small valley is apparent in the temporal history for drop radius from Figure 3, which can be traced to the microstructure developing around the drop (see Figure 2) during its initial swelling stage. (Note that such microstructure is absent from the numerical results of our previous study [5] but crops up in the additional results discussed below).

**Figure 4:**
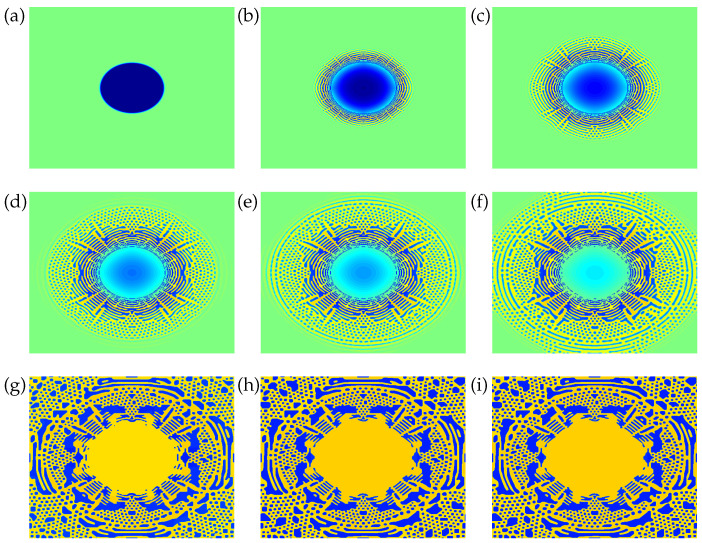
Snapshots of $x_{2}$ mole fraction at different (nondimensional) times (**a**) t=0, (**b**) t=0.085, (**c**) t=0.26, (**d**) t=0.54, (**e**) t=0.66, (**f**) t=0.96, (**g**) t=5.2, (**h**) t=22, (**i**) t=40 from phase-field simulation of an unstable drop (consisting of pure component 1) embedded in a two-component (2+3) liquid (with mole fractions $x_{2}=x_{3}=1/2$ at time zero) with NRTL parameters for a (1)benzene-(2)acetonitrile-(3)water mixture at T=300K (corresponding to point B in the phase diagram in Figure 1) on a $512^{2}$ grid. The initial radius of the drop is 40a.

In another simulation (case B), we chose a larger initial drop radius (40a) so that the global state of the system corresponds to point B in Figure 1, an unstable equilibrium state since the mixture at that location is within the spinodal region at that temperature. (The global composition for a mixture located at B is $x_{B}=\left(0.077,0.46,0.463\right)$.) Actually, for running case B we chose a different set of NRTL parameters corresponding to the same benzene-acetonitrile-water mixture as above at a smaller T=300K. As can be seen, at this smaller temperature, point B is well within the spinodal range (to a larger extent as compared to when T=333K). We ran our code with this initial condition and found a nontrivial dynamics of the concentration fields, in contrast to the simulation case discussed above, which can be completely characterized in terms of the drop size versus time dependence in Figure 3. In this case (see Figure 4), initially the radius of the drop increases with time as component 2 exits the continuous phase and enters into the drop. Furthermore, as the drop becomes larger and larger, a compositional disturbance (or wave) seems to propagate outwards from the center of the drop, converting an otherwise uniform composition [whose molar concentration was $x_{\infty }=\left(0,0.5,0.5\right)$ before passage of the disturbance] into a collection of nuclei as well as elongated drops. In fact, some of those single-phase microdomains that appeared as pseudospherical nuclei in our previous spinodal wave calculation [5] turn out to be elongated filaments in the NRTL-based calculation reported herein (see Figure 4). Apparently, after a number of coalescence events, a drop-like shape survives at the center of the box (with its radius tending to a constant value), surrounded by a complex filamentary pattern, while in our previous spinodal wave calculation [5] a single main drop survived at steady state at the center of the box, as all of the additional nuclei were slowly reabsorbed into the continuous phase.

## 5. Conclusions

We have shown results of 2D simulations at two different (uniform) temperatures of an isothermal phase-field ternary mixture model that incorporates NRTL as an excess free energy submodel in application to an isolated single-component (benzene) drop embedded in a continuous phase of a binary (acetonitrile+water) liquid as it grows or dissolves, depending on the global state of the system in the ternary phase diagram. We have reported a reabsorbing drop case wherein the global mixture composition corresponds to a stable location in the one-phase region of the phase diagram, so that the drop is bound to dissolve. This case can be characterized as nontrivial from a dynamical point of view, since it shows an increase-decrease behavior in the radius versus time dependence, similar to what had been observed from simulations of a ternary phase-field model that relied on the one-parameter Margules correlation as the excess Gibbs energy submodel [5]. With the NRTL-based ternary phase-field model discussed herein, we have also shown an unstable drop (i.e., corresponding to a global mixture composition within the spinodal range of the phase diagram) whose transient growth is accompanied by a spinodal wave (i.e., a compositional disturbance propagating outwards from the center of the drop), initially converting an otherwise uniform composition into a collection of small drops. However, at variance with our previous spinodal wave calculation (once again based on the one-parameter Margules correlation), in this NRTL-based calculation a number of pseudospherical nuclei (apparent in our previous calculation) are replaced by elongated filaments; consequently, at steady state, a drop-like shape survives at the center of the box, surrounded by a complex filamentary pattern.

## Figures and Tables

**Table 1:** NRTL parameters for the (1)benzene-(2)acetonitrile-(3)water ternary system at two different temperatures [28]. Note that $\Delta g_{ij}$ is in cal/mol.

Binary Parameter	300 K	333 K
$\Delta g_{12}$	693.61	998.2
$\Delta g_{21}$	92.47	65.74
$\Delta g_{13}$	3892.44	3883.2
$\Delta g_{31}$	3952.2	3849.57
$\Delta g_{23}$	415.38	363.57
$\Delta g_{32}$	1016.28	1262.4
$\alpha_{12}$	0.67094	0.88577
$\alpha_{13}$	0.23906	0.24698
$\alpha_{23}$	0.20202	0.3565

## Abbreviations

The following abbreviations are used in this manuscript:

UNIQUAC	UNIversal Quasi-Chemical
NRTL	Non-Random, Two-Liquid

## Appendix A. Hessian Determinant Based on the NRTL Equation

Based on Equation (13), the Hessian determinant needed to calculate the spinodal curves in Figure 1 is

(A1) $$\begin{array}{c} \Delta =\frac{\partial^{2}g^{th}}{\partial x_{1}^{2}}\frac{\partial^{2}g^{th}}{\partial x_{2}^{2}}-{\left(\frac{\partial^{2}g^{th}}{\partial x_{1}\partial x_{2}}\right)}^{2}=\left\{\frac{2x_{2}\left[G_{12}x_{1}\tau_{12}-G_{32}\left(x_{1}+x_{2}-1\right)\tau_{32}\right]{\left(G_{12}-G_{32}\right)}^{2}}{{\left[G_{12}x_{1}+x_{2}-G_{32}\left(x_{1}+x_{2}-1\right)\right]}^{3}}\right.\\ \left.-\frac{2x_{2}\left(G_{12}\tau_{12}-G_{32}\tau_{32}\right)\left(G_{12}-G_{32}\right)}{{\left[G_{12}x_{1}+x_{2}-G_{32}\left(x_{1}+x_{2}-1\right)\right]}^{2}}+\frac{2G_{13}\left[G_{13}\left(x_{2}-1\right)-G_{23}x_{2}\right]\tau_{13}}{{\left[\left(G_{13}-1\right)x_{1}+\left(G_{23}-1\right)x_{2}+1\right]}^{2}}\right.\\ \left.-\frac{2\left(G_{13}-1\right)\left[G_{13}\left(x_{2}-1\right)-G_{23}x_{2}\right]\left(G_{13}x_{1}\tau_{13}+G_{23}x_{2}\tau_{23}\right)}{{\left[\left(G_{13}-1\right)x_{1}+\left(G_{23}-1\right)x_{2}+1\right]}^{3}}+\frac{2G_{31}\left[G_{31}\left(x_{2}-1\right)-G_{21}x_{2}\right]\tau_{31}}{{\left[x_{1}+G_{21}x_{2}-G_{31}\left(x_{1}+x_{2}-1\right)\right]}^{2}}\right.\\ \left.+\frac{2\left(G_{31}-1\right)\left[G_{31}\left(x_{2}-1\right)-G_{21}x_{2}\right]\left[G_{31}\left(x_{1}+x_{2}-1\right)\tau_{31}-G_{21}x_{2}\tau_{21}\right]}{{\left[x_{1}+G_{21}x_{2}-G_{31}\left(x_{1}+x_{2}-1\right)\right]}^{3}}+\frac{1}{x_{1}}-\frac{1}{x_{1}+x_{2}-1}\right\}\\ \times \left\{\frac{2x_{1}\left[G_{21}x_{2}\tau_{21}-G_{31}\left(x_{1}+x_{2}-1\right)\tau_{31}\right]{\left(G_{21}-G_{31}\right)}^{2}}{{\left[x_{1}+G_{21}x_{2}-G_{31}\left(x_{1}+x_{2}-1\right)\right]}^{3}}-\frac{2x_{1}\left(G_{21}\tau_{21}-G_{31}\tau_{31}\right)\left(G_{21}-G_{31}\right)}{{\left[x_{1}+G_{21}x_{2}-G_{31}\left(x_{1}+x_{2}-1\right)\right]}^{2}}\right.\\ \left.+\frac{2G_{23}\left[G_{23}\left(x_{1}-1\right)-G_{13}x_{1}\right]\tau_{23}}{{\left[\left(G_{13}-1\right)x_{1}+\left(G_{23}-1\right)x_{2}+1\right]}^{2}}+\frac{2\left(G_{23}-1\right)\left(-x_{1}G_{23}+G_{23}+G_{13}x_{1}\right)\left(G_{13}x_{1}\tau_{13}+G_{23}x_{2}\tau_{23}\right)}{{\left[\left(G_{13}-1\right)x_{1}+\left(G_{23}-1\right)x_{2}+1\right]}^{3}}\right.\\ \left.-\frac{2G_{32}\tau_{32}}{G_{12}x_{1}+x_{2}-G_{32}\left(x_{1}+x_{2}-1\right)}-\frac{2{\left(G_{32}-1\right)}^{2}x_{2}\left[G_{32}\left(x_{1}+x_{2}-1\right)\tau_{32}-G_{12}x_{1}\tau_{12}\right]}{{\left[G_{12}x_{1}+x_{2}-G_{32}\left(x_{1}+x_{2}-1\right)\right]}^{3}}\right.\\ \left.-\frac{2\left(G_{32}-1\right)\left[G_{32}\left(x_{1}+2x_{2}-1\right)\tau_{32}-G_{12}x_{1}\tau_{12}\right]}{{\left[G_{12}x_{1}+x_{2}-G_{32}\left(x_{1}+x_{2}-1\right)\right]}^{2}}+\frac{1}{x_{2}}-\frac{1}{x_{1}+x_{2}-1}\right\}-\left\{\frac{G_{13}\tau_{13}}{\left(G_{13}-1\right)x_{1}+\left(G_{23}-1\right)x_{2}+1}\right.\\ \left.-\frac{G_{13}\left(G_{23}-1\right)\left(x_{1}+x_{2}-1\right)\tau_{13}}{{\left[\left(G_{13}-1\right)x_{1}+\left(G_{23}-1\right)x_{2}+1\right]}^{2}}+\frac{G_{23}\tau_{23}}{\left(G_{13}-1\right)x_{1}+\left(G_{23}-1\right)x_{2}+1}-\frac{\left(G_{13}-1\right)G_{23}\left(x_{1}+x_{2}-1\right)\tau_{23}}{{\left[\left(G_{13}-1\right)x_{1}+\left(G_{23}-1\right)x_{2}+1\right]}^{2}}\right.\\ \left.-\frac{\left(G_{13}-1\right)\left(G_{13}x_{1}\tau_{13}+G_{23}x_{2}\tau_{23}\right)}{{\left[\left(G_{13}-1\right)x_{1}+\left(G_{23}-1\right)x_{2}+1\right]}^{2}}-\frac{\left(G_{23}-1\right)\left(G_{13}x_{1}\tau_{13}+G_{23}x_{2}\tau_{23}\right)}{{\left[\left(G_{13}-1\right)x_{1}+\left(G_{23}-1\right)x_{2}+1\right]}^{2}}\right.\\ \left.+\frac{2\left(G_{13}-1\right)\left(G_{23}-1\right)\left(x_{1}+x_{2}-1\right)\left(G_{13}x_{1}\tau_{13}+G_{23}x_{2}\tau_{23}\right)}{{\left[\left(G_{13}-1\right)x_{1}+\left(G_{23}-1\right)x_{2}+1\right]}^{3}}-\frac{\left(G_{21}-G_{31}\right)G_{31}x_{1}\tau_{31}}{{\left[x_{1}+G_{21}x_{2}-G_{31}\left(x_{1}+x_{2}-1\right)\right]}^{2}}\right.\\ \left.+\frac{G_{31}\tau_{31}-G_{21}\tau_{21}}{x_{1}+G_{21}x_{2}-G_{31}\left(x_{1}+x_{2}-1\right)}+\frac{\left(G_{31}-1\right)x_{1}\left(G_{31}\tau_{31}-G_{21}\tau_{21}\right)}{{\left[x_{1}+G_{21}x_{2}-G_{31}\left(x_{1}+x_{2}-1\right)\right]}^{2}}\right.\\ \left.+\frac{\left(G_{21}-G_{31}\right)\left[G_{21}x_{2}\tau_{21}-G_{31}\left(x_{1}+x_{2}-1\right)\tau_{31}\right]}{{\left[x_{1}+G_{21}x_{2}-G_{31}\left(x_{1}+x_{2}-1\right)\right]}^{2}}\right.\\ \left.+\frac{2\left(G_{31}-1\right)\left(G_{31}-G_{21}\right)x_{1}\left[G_{31}\left(x_{1}+x_{2}-1\right)\tau_{31}-G_{21}x_{2}\tau_{21}\right]}{{\left[x_{1}+G_{21}x_{2}-G_{31}\left(x_{1}+x_{2}-1\right)\right]}^{3}}-\frac{\left(G_{12}-G_{32}\right)G_{32}x_{2}\tau_{32}}{{\left[G_{12}x_{1}+x_{2}-G_{32}\left(x_{1}+x_{2}-1\right)\right]}^{2}}\right.\\ \left.+\frac{G_{32}\tau_{32}-G_{12}\tau_{12}}{G_{12}x_{1}+x_{2}-G_{32}\left(x_{1}+x_{2}-1\right)}+\frac{\left(G_{32}-1\right)x_{2}\left(G_{32}\tau_{32}-G_{12}\tau_{12}\right)}{{\left[G_{12}x_{1}+x_{2}-G_{32}\left(x_{1}+x_{2}-1\right)\right]}^{2}}\right.\\ \left.+\frac{\left(G_{12}-G_{32}\right)\left[G_{12}x_{1}\tau_{12}-G_{32}\left(x_{1}+x_{2}-1\right)\tau_{32}\right]}{{\left[G_{12}x_{1}+x_{2}-G_{32}\left(x_{1}+x_{2}-1\right)\right]}^{2}}\right.\\ {\left.+\frac{2\left(G_{32}-1\right)\left(G_{32}-G_{12}\right)x_{2}\left[G_{32}\left(x_{1}+x_{2}-1\right)\tau_{32}-G_{12}x_{1}\tau_{12}\right]}{{\left[G_{12}x_{1}+x_{2}-G_{32}\left(x_{1}+x_{2}-1\right)\right]}^{3}}+\frac{1}{x_{1}+x_{2}-1}\right\}}^{2} \end{array}$$
